# Bevacizumab-Associated Bowel Perforation Causing Evisceration

**DOI:** 10.7759/cureus.98021

**Published:** 2025-11-28

**Authors:** Maziar Fazel Darbandi, Garrett Johnson, Krista Hardy, Ashley Vergis

**Affiliations:** 1 Department of Surgery, University of Manitoba, Winnipeg, CAN

**Keywords:** bevacizumab therapy, bowel evisceration, metastatic rectal cancer, parastomal hernia, spontaneous bowel perforation

## Abstract

Bevacizumab is a monoclonal antibody that targets vascular endothelial growth factor (VEGF) and inhibits angiogenesis in solid organ tumors. Its use in conjunction with fluorouracil-based chemotherapy for the management of metastatic colorectal cancer (CRC) has proven effective. There is a known, rare chance of gastrointestinal perforation associated with its use.

We present a case of a man in his 70s on bevacizumab for metastatic rectal cancer, who presented to the hospital with small bowel evisceration through perforation at his colostomy site, ostensibly caused by a chronic parastomal hernia and bevacizumab chemotherapy. He required emergent surgery. After undergoing resection of an ischemic segment of small bowel, the perforated segment of his colon, and primary repair of his parastomal hernia, and fashioning of a new end colostomy, he recovered well and left the hospital two weeks later. After discharge from the hospital, he was able to continue with palliative chemotherapy.

## Introduction

Colorectal cancer (CRC) is the third most commonly diagnosed cancer worldwide and the second most common cause of cancer-related death [1]. Up to 20% to 25% of patients diagnosed with CRC are found to have synchronous metastases, most commonly in the liver [2,3]. In these cases, the role for surgery may be limited, and patients require systemic therapy for management of their cancer.

Bevacizumab was one of the first targeted therapy agents, with Hurwitz et al. publishing their clinical trial comparing fluorouracil-based chemotherapy plus bevacizumab to chemotherapy alone in patients with metastatic CRC in 2004 [4]. The trial showed that there was improvement in both median duration of survival and disease-free survival in patients with metastatic CRC who had bevacizumab in conjunction with fluorouracil-based chemotherapy [4].

Bevacizumab acts by inhibiting angiogenesis, which is a hallmark of solid organ tumors. It does so by binding to all circulating, soluble vascular endothelial growth factor-A (VEGF-A) isoforms [3-6]. By binding the molecule, it prevents its binding to the VEGF receptor, thereby inhibiting the proliferation of new blood vessels [3-6].

Although a relatively well-tolerated medication, bevacizumab does have a wide array of potential side effects. Most commonly, hypertension, bleeding, pulmonary hemorrhage, proteinuria, arterial thrombotic events, wound healing complications, fistula formation, and gastrointestinal perforations, among others [4-10]. Due to complications with wound healing, it is recommended for patients who receive bevacizumab that the time interval to operation exceed four weeks to avoid increased risk for mortality [11]. Furthermore, patients who had a bevacizumab infusion within 28 days of emergency surgery had an increased risk of mortality. Compared to controls, patients on bevacizumab had a three-fold increase in their risk for developing gastrointestinal perforation, which could be life-threatening [10,12-13].

## Case presentation

A gentleman in his 70s with a past medical history of benign prostatic hyperplasia, irritable bowel syndrome, depression, and prior inguinal hernia repair had originally presented to the hospital with bright red blood per rectum. Thereafter, endoscopy revealed a rectal mass at the first rectal fold, with biopsies proving a diagnosis of invasive adenocarcinoma. Unfortunately, his staging workup revealed metastases to both the liver and lungs. Two months after diagnosis, he underwent a diverting loop sigmoid colostomy due to impending large bowel obstruction stemming from his rectal cancer. Two months after diversion, once he had recovered from his surgery, he was started on folinic acid, fluorouracil, and irinotecan (FOLFIRI) and bevacizumab for palliative chemotherapy. Complications of initiating treatment included a pulmonary embolism and atrial fibrillation, and he was started on apixaban and diltiazem for management of these conditions. Three months after being started on palliative treatment, he developed a large parastomal hernia. He was offered repair but opted to continue watchful waiting, as he was minimally symptomatic and did not want to interrupt systemic therapy. The patient had stable disease for months, with no progression of his metastatic disease and some improvement in the burden of hepatic metastases.

**Figure 1:**
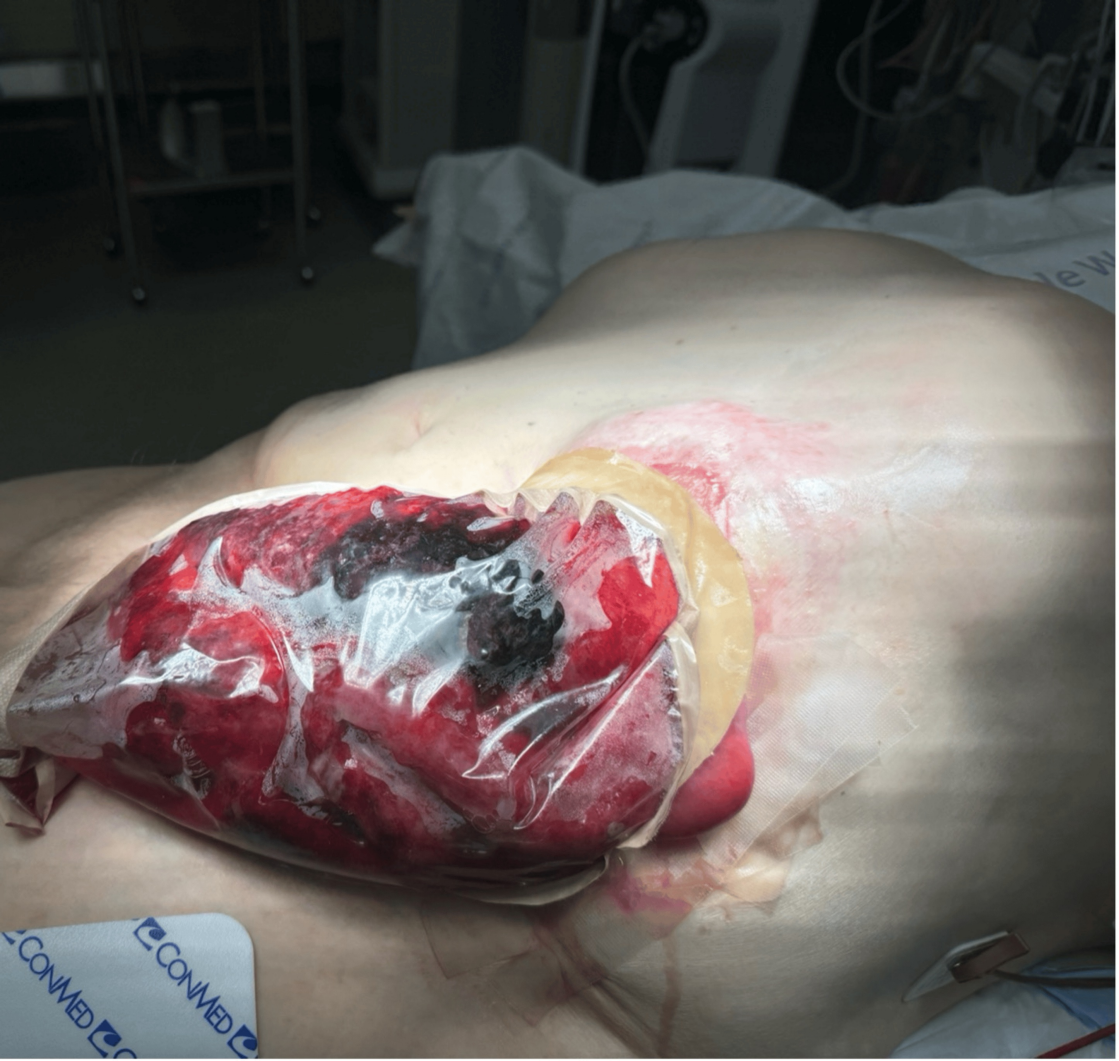
Eviscerated bowel within a plastic bag when the patient assessed

One day, about eight months after starting treatment and five months after developing the parastomal hernia, the patient noticed what he described as “stoma prolapse” into his ostomy appliance, which was aggravated by a vigorous bout of nausea and emesis. He had received cycle 16 of FOLFIRI plus bevacizumab two days prior, and it was normal for him to have nausea and emesis for a few days after each dose. However, on this day, he had numerous bouts of unrelenting nausea and emesis with significant fatigue thereafter. After waking up from a nap, he noticed what appeared to be small bowel filling his ostomy appliance. He quickly presented to the emergency department, where he was in stable condition, alert, oriented, and endorsed no significant pain or ongoing nausea and emesis. Upon removal of his stoma appliance, it was evident that there was a small bowel protruding through the lumen of his colostomy, which exhibited ischemic changes (Figure 1).

**Figure 2:**
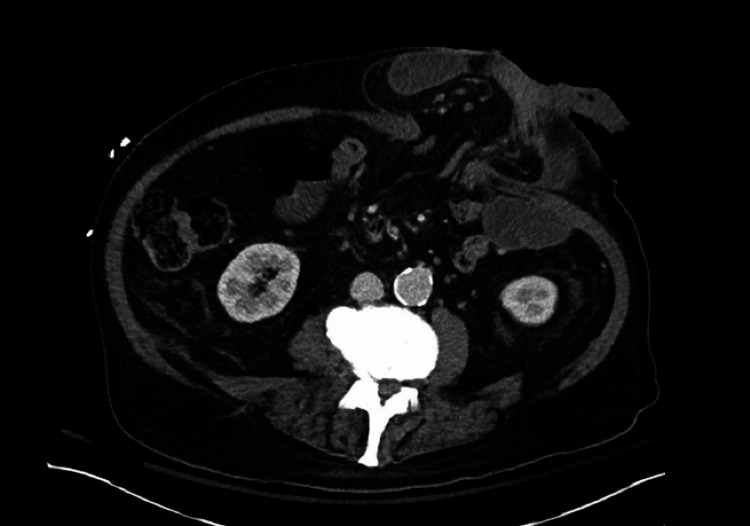
CT scan at presentation

**Figure 3:**
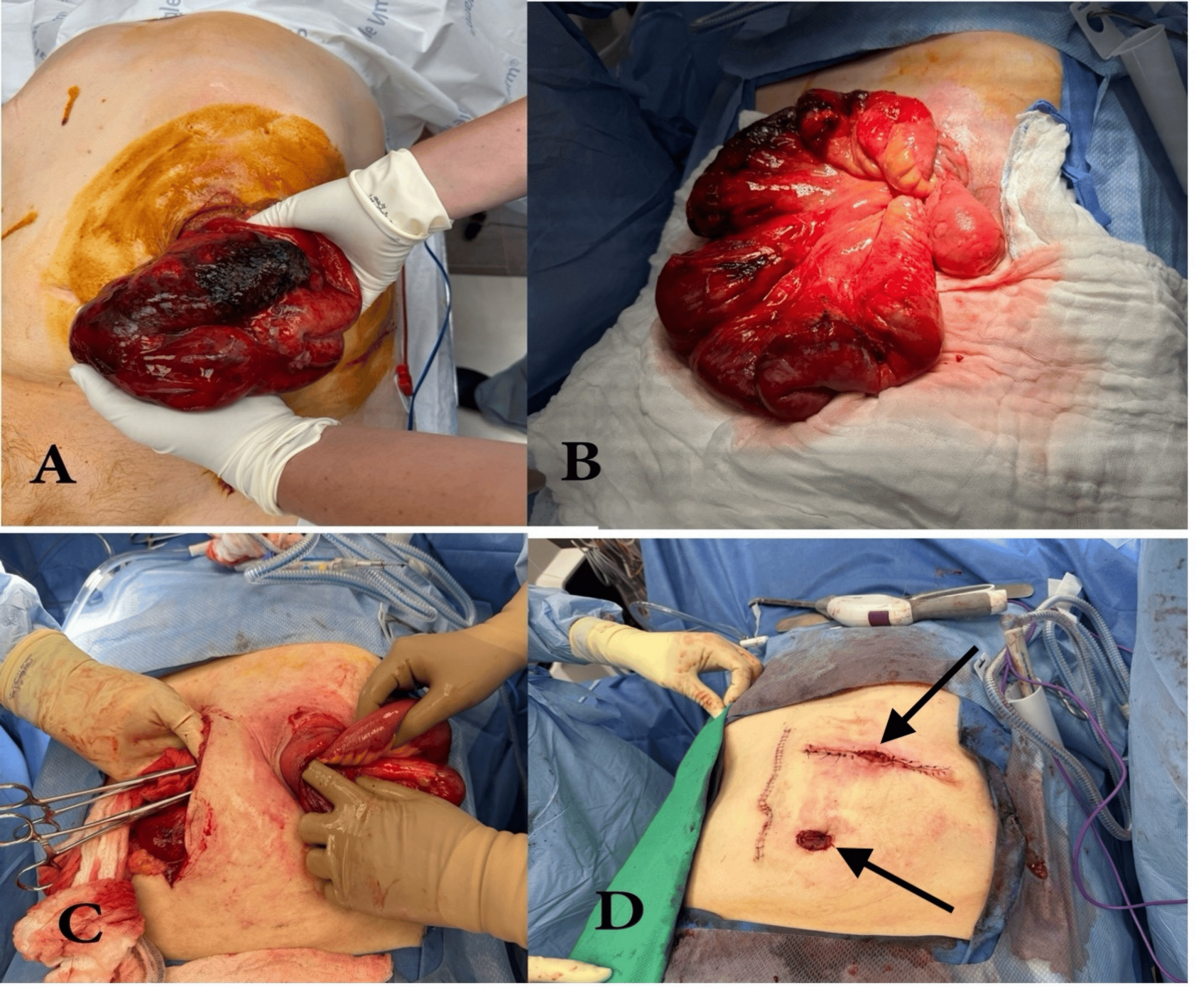
Intraoperative findings, management of bowel perforation and evisceration, and repair of parastomal hernia. A: Area of ischemic changes was evident while the abdomen was being prepped. B: Exploring the cause of evisceration. C: Midline laparotomy was performed, and the eviscerated bowel was reduced. The surgeon’s left-hand index finger was in the colostomy ulcer through which the small bowel had eviscerated. D: Midline laparotomy was closed, the hernia repaired, the mucous fistula brought up (arrow indicating left lower quadrant), and the end colostomy created (arrow indicating left upper quadrant) with the surrounding skin defect revised.

The patient was transferred to another center for surgical evaluation. He underwent cross-sectional imaging (Figure 2) as he was stable to aid with operative planning. The patient underwent emergent surgery (Figure 3). A midline laparotomy was performed, and the abdomen was explored. The ostomy, along with the eviscerated bowel, was reduced. There was a short segment of ischemic and necrotic small bowel, which was resected, located 15 cm proximal to the terminal ileum and 200 cm distal to the ligament of Treitz. The remaining small bowel was reconstructed via a standard primary stapled side-to-side antiperistaltic small bowel anastomosis. The hernia sac was dissected out circumferentially. The internal and external oblique muscle layers were delineated and separated. Both muscle layers were reapproximated and closed primarily with interrupted polypropylene sutures, leaving just enough room for an end sigmoid colostomy. Due to the associated contamination, no mesh was placed. A distal mucous fistula was matured through a separate lower abdominal incision. The abdomen was irrigated thoroughly with warm saline. The midline fascia was closed with a running #1 polydioxanone suture, and the skin was closed with staples.

The patient was deemed ready for discharge on postoperative day 11; his stay was prolonged due to ileus requiring nasogastric tube decompression and bowel rest, subsequently followed by high-output ileostomy requiring medical management to resolve. The pathology report showed hemorrhagic congestion of the small bowel. Along with perforation, transmural inflammation, hemorrhage, edema, fibrosis, and serositis of the colostomy, which had been resected. He was started back on his palliative chemotherapy one month after discharge home and is currently starting cycle 34 with stable disease, 18 months since starting treatment.

## Discussion

Gastrointestinal perforation is a documented rare side effect of bevacizumab use. Case reports and series have been published on this topic, with the mechanism thought to be secondary to ischemic changes in the bowel wall [6, 11-17]. Proposed mechanisms for ischemia to the bowel wall include thrombosis or vasoconstriction of the splanchnic or mesenteric vessels [10, 13-15]. Another interesting case documented the incidence of pneumatosis intestinalis, which luckily did not lead to perforation and resolved after six weeks of stopping bevacizumab [18]. Bevacizumab is also known to cause issues with tissue and wound healing and is thought to increase the risk for developing incisional hernias [6, 19].

To our knowledge, this is the first case in the literature where a patient presented with small bowel evisceration through the site of spontaneous perforation at a colostomy. Ostensibly, chronic pressure caused by the parastomal hernia, combined with wound healing difficulties from bevacizumab, led to this unique presentation.

Our case highlights the outcomes of both poor wound healing and gastrointestinal perforation secondary to treatment with bevacizumab. This reinforces the need for caution in this medication’s use, especially in patients who already exhibit side effects such as poor wound healing, thrombosis, and cardiac side effects, as in our patient.

The incidence of parastomal hernia in patients with a loop colostomy can be up to 30% and can increase with time [20]. Importantly, as patients’ lives are prolonged by advances in systemic therapy, these complications could become more prevalent.

Gastrointestinal perforation while on bevacizumab is more common in patients with CRC, cervical cancer, and prostate cancer; however, no other risk factors have been identified that could help predict who may be at higher risk [11-13]. Therefore, it may be futile to aim to predict who may suffer from these complications.

Gastrointestinal perforation from any cause can be life-threatening; in the setting of bevacizumab use, case reports and studies have shown the potential for devastating outcomes [6-8, 11-17]. Our patient benefited from early recognition and emergent action and definitive management before he deteriorated. Interestingly, one could posit that because his bowel eviscerated and his perforation was not confined to his abdomen, this may have spared him from intra-abdominal sepsis and contributed to his relatively stable status.

This case provided challenging intraoperative decision-making, such as the decision to perform a small bowel anastomosis and risk a leak versus an end ileostomy, which, in the setting of chemotherapy, could prove difficult to manage with high outputs [21]. These could both lead to the patient no longer being fit for systemic therapy. Moreover, the decision-making around how to best manage the patient’s hernia was quite complex. The World Society of Emergency Surgery 2017 guidelines state that for contaminated dirty wounds, primary repair is effective for defects less than 3 cm in size, and where suture-based repair is not feasible, mesh-based repair with a biologic or polyglactin mesh, or open wound management with delayed repair, is appropriate [22]. This is further compounded by the fact that the patient had already proven to suffer from poor wound healing.

## Conclusions

Although rare, patients on bevacizumab are at risk for gastrointestinal perforation, and healthcare providers must maintain a high index of suspicion. As systemic therapy improves in its effectiveness to prolong life in patients with metastatic CRC, the occurrence of rare complications may become more common. This case highlights the importance of prompt intervention to ensure good outcomes and avoid mortality in these patients. Fortunately, our strategies were successful, and the patient recovered well and was later discharged home.
